# 

**Published:** 1864-01

**Authors:** 


### CONTENTS.

### ORIGINAL CONTRIBUTIONS.

ART. I. The Organs of Sensation and Motion Discovered. By W. Byrd Powell, M.D., of Covington, Ky., ..... 1
ART. II. Singular Freak in Nature, or Malformation. By D. O. Crist, M.D., of Bloomington, Ill., ..... 13
ART. III. An Epidemic of Erysipelas, caused by the Decomposition of Animal Matter in Chicago River. By E. Andrews, M.D., Professor of Surgery in Chicago Medical College, ..... 17
ART. IV. Cases Read before the Chicago Medical Society. By Hiram Wanzer, M.D., of Chicago, Ill., ..... 21
ART. V. Clinical Reports. By N. S. Davis, M.D., Prof. of Practical and Clinical Medicine, ..... 26

### SELECTIONS.

Velocity of Nervous Force, ..... 33
The Surgical Treatment of Amenorrhœa. By Horatio R. Storer, M.D., of Boston, Surgeon to the Pleasant Street Hospital for Women, ..... 34
Latent Syphilis, ..... 44
Variococele of Twelve Years' Duration; Great Pain in the Testicle; Operation by Wire Ligature; Cure, ..... 46

### EDITORIAL.

Small-Pox Hospital, ..... 48
United States Sanitary Commission, ..... 51
The Medical Staff of England, ..... 54
On the Value of Tincture of Iodine as a Test for Diabetic Urine, ..... 54
Letter from New York, the new (or old) Anæsthetic, ..... 55
Chicago Medical Journal, ..... 58
Glycerine, ..... 58
Hæmostasis, ..... 61
Test for Sulphur, ..... 61

